# LTCC Flow Sensor with RFID Interface

**DOI:** 10.3390/s20010268

**Published:** 2020-01-02

**Authors:** Mariusz Węglarski, Piotr Jankowski-Mihułowicz, Grzegorz Pitera, Dominik Jurków, Mateusz Dorczyński

**Affiliations:** 1 Department of Electronic and Telecommunications Systems, Rzeszów University of Technology, Wincentego Pola 2, 35-959 Rzeszów, Poland; grzesiekpitera@wp.pl; 2 Faculty of Microsystem Electronics and Photonics, Wrocław University of Technology, Janiszewskiego 11/17, 50-372 Wrocław, Poland; dominik.jurkow@wp.pl (D.J.); mateusz.dorczynski@gmail.com (M.D.)

**Keywords:** RFID, LTCC, energy harvesting, intelligent sensor, RFID tag, semi-passive transponder, thick-film sensor, wireless sensor network

## Abstract

The idea of battery-less flow sensors and their implementation in wireless measurement systems is presented in this research article. The authors take advantage of their latest achievements in the Low Temperature Co-fired Ceramic (LTCC) technology, RadioFrequency Identification (RFID) technique, and increasing availability of low power electronics in order to get rid of the need to use electrochemical cells in a power supply unit of the elaborated device. To reach this assumption, special care has to be put on the energy balance in such an autonomous sensor node. First of all, the new concept of an electromagnetic LTCC turbine transducer with a signal conditioner which only draws a current of around 15 µA, is proposed for measuring a flow rate of fluids. Next, the autonomy of the device is showed; measured data are gathered in a microcontroller memory and sent to a control unit via an RFID interface which enables both information exchange and power transfer. The energy harvested from the electromagnetic field is used to conduct a data transmission, but also its excess can be accumulated, so the proposed sensor operates as a semi-passive transponder. The total autonomy of the device is achieved by implementing a second harvester that continually gathers energy from the environmental electromagnetic field of common active radio systems (e.g., Global System for Mobile Communications (GSM), wireless network Wi-Fi).

## 1. Introduction

When there is a necessity to apply measuring equipment in a harsh environment, in hardly accessible cases or when installation of a wire system is unprofitable, then autonomous sensor nodes with a wireless interface can be used [1,2]. They are able to measure physical quantities or monitor surrounding conditions and exchange data with a control unit by the wireless medium. The crucial disadvantage of the contemporary intelligent data acquisition nodes is the necessity to use electrochemical cells as an energy source. With growing demand for implementing such systems, a severe problem arises with the maintenance of sensor activity over a long period of time, and the utilization of old waste batteries. Sometimes the problem is overcome by supplying electronic circuits with energy gathered from solar radiation, heat flow, or mechanical vibration [3,4], but these resources cannot always be used. Since the contemporary semiconductors consume increasingly less energy, it is possible to supply the sensor node by the electromagnetic field, but special care has to be put on the energy balance in its circuitry. The hardware components of such an electronic system has to be highly power-efficient because energy conveyed by the electromagnetic field is very low, e.g., harvesting from Global System for Mobile Communications (GSM) operating at 0.9 GHz or 1.8 GHz it is possible to obtain up to 0.1 µW/cm^2^, from the wireless network Wi-Fi 2.4 GHz up to 0.01 µW/cm^2^ which is a very low value as compared to the light source, 10 mW/cm^2^, vibration, 0.1 mW/cm^2^ or heat, 10 mW/cm^2^ [3,4].

In order to achieve the new design of the flow sensor that could meet the requirements of modern measuring systems, several improvements need to be made according to previous authors’ projects [5,6] or other constructions known in this field [7,8,9]. First of all, a RadioFrequency Identification (RFID) interface is implemented instead of a typical wireless communication (Section 2.1). The transmission drivers operating in common standards such as Bluetooth (IEEE 802.15.1), Zigbee (IEEE 802.15.4), Wi-Fi (IEEE 802.11), etc. have to be supplied with significant amount of energy to generate radio waves when sending data. Then they work as typical conventional Short Range Devices (SRD) [10]. On the other hand, the RFID transmitter (for example in passive transponders) by nature does not require any additional supply in order to pass information [11].

Since the battery-less electronics suffer from a lack of energy, the next step in designing the modern flow sensor is to elaborate an ultra low power transducer (Section 2.2). For this purpose, new concepts of an electromagnetic turbine fabricated in Low Temperature Co-fired Ceramic (LTCC) and a signal conditioning circuit which only draws a current of around 15 µA are elaborated in order to measure the flow rate of fluids. Additionally, significant improvements were implemented in the manufacturing process (Section 3), allowing the authors to eliminate some of the problems often encountered in LTCC technology [12,13].

If the passive transponder was equipped with additional sensing features it could operate as a battery-less sensor node. But there are some technical problems when designing such a solution. Although it is achievable to find very rare passive RFID chips with the possibility to share the energy with additional internal function blocks [14,15], the measuring procedures cannot be executed without a working Read/Write Device (RWD); transponders are activated only in the electromagnetic field generated by the RWD antenna. Moreover, only sensors that measure the temperature or voltage of the supply unit can be created on the basis of the mentioned chip. The full autonomous sensor node that can measure any physical quantity can be designed only after applying a battery source and using chips with a semi-passive mode. Such a model is implemented for example in the Wireless Identification and Sensing Platform (WISP) proposed by Intel Research Seattle laboratory [16] or Self-Powered Augmented RFID Tag for Autonomous Computing and Ubiquitous Sensing (SPARTACUS) [17], and other research systems [18,19]. These untypical solutions operate as semi-passive transponder-sensors with an internal analog-to-digital converter and can measure different physical quantities (humidity [19,20], temperature [19,21,22], light intensity [21,22], pressure [23], acceleration [24], gas [19,25], etc.). In all examples, the devices are supported by an intelligent microcontroller for acquiring data and managing the systems. 

Since the semi-passive transponder has a radiofrequency front-end powered from the electromagnetic field, it is equipped with a supply block consisting of an electrical harvester integrated with an advanced low voltage regulator. If the chip provides an additional voltage output, a supercapacitor can be used instead of a battery, but the electromagnetic field generated by the RWD is a low-yield and uncertain source for recharging the supercapacitor. It is a good idea to support it by electric energy obtained from mechanical, thermal, or solar transducers [26,27]. Nevertheless, the electromagnetic field generated by the typical radiocommunications systems (e.g., base stations of GSM, Wi-Fi, Worldwide Interoperability for Microwave Access—WiMAX, etc.) that are always present almost everywhere on the Earth is a more natural source, especially in the view of RFID system operation principles and technological aspects of device miniaturization. Although the semi-passive transponder-sensor operating autonomously outside the Interrogation Zone (IZ) [11] can be powered by an environmental electromagnetic field, this type of supply source is very weak, and problems of harvesting, storing, and converting very low energy to a usable level has to be solved. In order to cope with these drawbacks the authors propose the special construction of the RFID semi-passive transponder-sensor with a two-antenna system (Section 4).

## 2. Principles of Experiment

### 2.1. Autonomous RFID Transponder-Sensor

**Figure 1:**
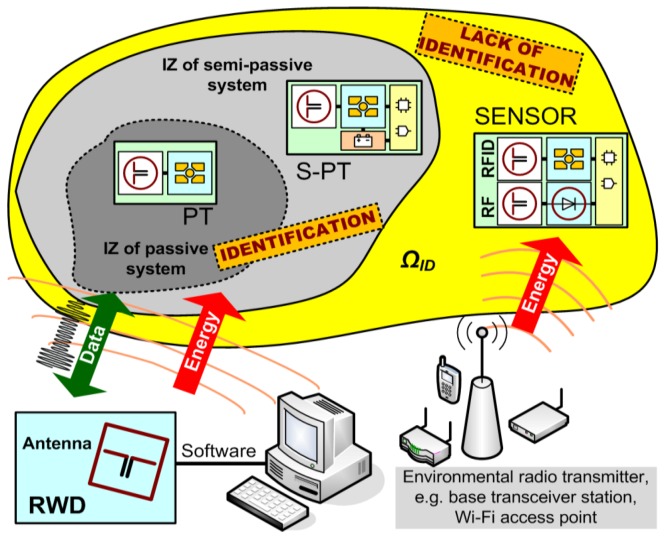
Block diagram of RadioFrequency Identification (RFID) system with autonomous RFID sensor node. IZ—interrogation zone; *Ω_ID_*—work space of user application; PT—passive transponders, S-PT—semi-passive transponder, SENSOR—Semi-passive transponder-sensor; PT, S-PT, SENSOR are compared in Table 1.

In general, RFID systems consist of a read/write device that includes a transmitter/receiver unit with connected antenna and at least a one electronic transponder (often called electronic tag) that is intended for marking an object (Figure 1). A typical RFID passive tag contains a chip attached to an antenna and harvests energy from the electromagnetic field generated by the RWD antenna. The accumulated energy is used for automatic identification, that is for passing the Unique Identification Number (UID) and other data allowed by the transmission protocol. The UID is permanently assigned to the tag. The communication process can be activated only when transponders are in the interrogation zone [11,14] (IZ is the most important parameter that describes the performance of RFID systems, comprehensively covering energy and communication conditions that have to be met in order to establish efficient communication link), which means that radiofrequency front-end is powered by energy harvested from the electromagnetic field. In the RFID systems, the back-scattering modulation is used, so transponders only reflect the waves back to the RWD; it does not emit any energy, as in the case of conventional SRDs [10]. Signal propagation consists in changing the input impedance in the antenna front-end of the RFID chip which influences the electromagnetic field and then is recognized by the RWD. 

Although the RFID technique is dedicated to performing automatic identification, but according to the newest trends (the third stage of development in automatic identification systems [28]), the RFID tags should provide besides details about marked objects (i.e., unique identification number, producer’s serial number, and information about product), also extended variable data gathered from surroundings by built-in sensors of different physical quantities. The measuring facilities can be easily implemented in RFID semi-passive transponders [14,15,16,17,18,19,20,21,22,23,24,25] where the chip is supported by a battery supply. Principles of the communication between the semi-passive tags and the RWD are the same as in the previously described passive model. The antenna circuit, memory, and digital control system responsible for data transmission are always powered by the electromagnetic field generated by the RWD. Thus, the extra power source supplies an internal circuit of the chip including additional functional blocks (e.g., analog-to-digital converter, extended memory of user data, drivers of wire serial data transmission, thermal or voltage transducers, and other integrated circuits) that are not directly related to the automatic identification process. It can be even used for supplying external integrated circuits (e.g., microcontrollers) of autonomous sensor nodes. Moreover, since the internal electronics are powered by the battery, it does not load the energy harvester of the tag radiofrequency front-end and therefore the sensitivity of the chip increases [14].

The autonomous transponder without battery can be built on the basis of the aforementioned RFID chip with an internal memory of application data and a serial interface for communication with a surrounding circuitry. When the chip is provided with a power output, the supercapacitor can be applied in order to supply the sensor node outside the IZ. Nevertheless, the additional harvester can be used to gather extra energy from the environment, e.g., the electromagnetic field generated by typical radiocommunications systems [14,15].

The comparison between ideas of passive and semi-passive transponders is presented in Figure 1 and main characteristics are summarized in Table 1. The battery-free semi-passive RFID transponder-sensor proposed in the paper is designed to operate autonomously outside the interrogation zone, although it should still be considered an element of the automatic identification system. It is equipped with an additional harvester that gathers extra energy from the electromagnetic field of a common telecommunication system—GSM900. The supporting power source is used to charge the supercapacitor and to further supply the microprocessor measurement unit and the amplifier dedicated for conditioning signal from the turbine gas flow transducer. In consequence, the flow sensor can operate and acquire samples outside the IZ. It should be noted that it is not possible to utilize energy from the RFID system because the designed voltage regulator has a simplified but energy efficient electrical circuit. This energy is only used for communication purposes. Nevertheless, since the SL900A chip provides an output voltage from the internal harvester, it is possible to apply it to power the flow sensor, but the regulator has to be redesigned and expanded, which will be done in the next stage of research.

Although the measured data are processed and saved inside the implemented microcontroller, they cannot be retrieved in the RFID system straight from the microprocessor memory because of the described scheme of power distribution and the fact that there is no possibility to access this memory by the RFID interface. But the Serial Peripheral Interface (SPI) is built into the SL900 chip. Thus, the pre-processed acquired values in the form of floating point numbers are written to the RFID chip memory by the SPI as two-byte data. In this manner, the saved information is accessible by the RWD if only the sensor appears in the IZ. 

### 2.2. Low Power LTCC Flow Sensor

The low temperature co-fired ceramic technology is frequently used for fabricating hybrid microelectronic devices [13,29,30]. The main advantage of LTCC-based hybrids is high environmental resistance. They are resistant to most chemical substances and against high temperature. Additionally, the ceramics have inherent properties such as good mechanical strength, thermal stability, and dielectric parameters (resistivity, relative permittivity and loss tangent) that make them perfect for packaging, three-dimensional (3D) structuring, RF signal integrity, etc. The possibility of integrating electronic wirings, magnetic or piezoelectric components, and thick-film passive elements in 3D mechanical structures complements the benefit array of this technology. Therefore, LTCC is widely used in environmental resisted, multilayer, high frequency circuits, sensors, actuators, micro-systems, etc [8,31,32,33]. Since micro-fluidic channel and chamber systems together with actuators, sensors, and electronic circuits can be bound in a common multilayer structure, the great opportunity to create sophisticated platforms for micro-reactors appears. Such devices can be used in applications of the chemical engineering, biotechnological, medical, and pharmaceutical industries, environmental pollution sensing, etc. [34,35,36].

The standard LTCC flow sensors operate on the basis of thermal effects [8,9]. A great advantage of this solution is its high reliability. But, the amount of energy that is consumed by an internal heater means that this construction is not used in a sensor node without a battery. Significantly less energy is dissipated by a LTCC sensor with a turbine [5,7]. The first rotating turbine fabricated in the LTCC technology with Sacrificial Volume Materials (SVM) was presented by Peterson at al. [7]. A moving component was only shown by the author and information about any detection of the gas flow velocity was not provided in this paper. Two types of sacrificial materials may be used in the 3D structuration of cavities and internal channels: fugitive (FSM) and mineral (MSM) materials. The first group is based on carbon, wax, or polymer compositions, and they evaporate during sintering [34]. When sacrificial materials are laminated or disappearing in a firing process, the ceramic layers may sag, and thus the designed dimensions of cavities change. In addition, a well-characterized ramping profile of temperature has to be applied at the sintering stage in order to allow SVM to burn out completely, and to avoid residual clogging of micro-channels. As a consequence, it is very hard to obtain spatial components with high precision. To cope with this problem, the MSM can be used, but then a post-firing chemical or mechanical process of removing it has to be applied. The SVMs also cause additional struggles like dealing with a shrinkage mismatch or chemical inter-reaction during sintering at high temperature. As a solution, to avoid these disadvantages, the use of lamination adhesives and progressive lamination is reported in the recent literature [35]. In this case, the high binding quality is achieved at moderate values of pressure and temperature at the expense of processing additional linking layers. Moreover, the final multilayer structure may suffer from a delimitation effect under a high pressure forced into the internal cavities. 

One of the approaches to achieve the goal of the authors’ research without the SVMs is to design the transducer as a combination of the operation chamber in the LTCC and the cover made of ceramic, transparent glass or even polymer [36]. This manner is frequently use in applications involving optical detection of fluid behavior. By coating LTCC structure with glaze layer, it is possible to obtain irreversible bonding between the both parts [37]. Further improvement can consist in applying the laser technique to form different shapes in multilayer structures (especially in the chamber part). The most sophisticated seems to be the laser ablation in which an infrared beam is used to precisely ablate surface of an unfired LTCC stack of green tapes [33]. The depth of ablation depends on applied process parameters like power, head speed, pulse repetition rate, etc. Since the ceramic sheets are soft, they can be damaged, thus patterning micro-components is a challenging technique in this case. Moreover, the laser beam has low depth-of-field, thus it has to be refocused and the cutting cycles have to be repeated in order to achieve maximum precision as well as smooth edges and surfaces. On the other hand, a single sheet can easily be cut with the laser, but then the very troublesome cycles of stacking small elements has to be involved. 

The use of laser cutting and thermo-compressive lamination in manufacturing the LTCC turbine gas flow sensor was reported by Jurków at all. [5]. The author applied an optoelectronic system for estimating the measured values. Unfortunately, the proposed technology consisting in the laser cutting and precise lamination of several very small components is very difficult, especially from the roll-to-roll fabrication and tapes misalignment point of view. Additional problem is with the mentioned integration of transparent glass (utilized for providing light inside the sensor chamber) with the LTCC substrate. Due to the technological imperfection, the reliability of the developed device is very low—the rotor often gets stuck inside the turbine chamber. Moreover, despite the proposed optoelectronic flow sensor with turbine consumes very small amount of energy, it still cannot be supplied by the electromagnetic field of the environment. In the electronic circuit there are elements such as a Light-Emitting Diode (LED) that dissipate too much energy (e.g., supply current up to 1 mA is needed for powering energy-efficient LED diode). 

The attempt to reach lower energy consumption is the reason why the authors developed the new flow transducer based on electromagnetic induction phenomena. The transducer itself does not load the supply source in semi-passive RFID transponder-sensor, it is the source of a weak voltage signal that has to be amplified for measurements. Since the transparent cover does not have to be implemented in the new design, the authors could slightly simplify the technological process of manufacturing the transducer. Admittedly, the mechanical machining is often used in the LTCC devices fabrication, and the precision of drilling or milling tools is comparable to the laser treatment. The most common method is punching in an unfired single green sheet [38]. To obtain the chamber component with a sufficient accuracy, the authors have used a Computer Numerically Controlled (CNC) routing machine. In this way, any troubles with sheet misalignment during stacking or surface roughness in the laser ablation are prevented, and fine edges of the cut unfired LTCC stack are provided. 

## 3. Electromagnetic LTCC Turbine Flow Sensor

### 3.1. Idea of Novel Construction

**Figure 2:**
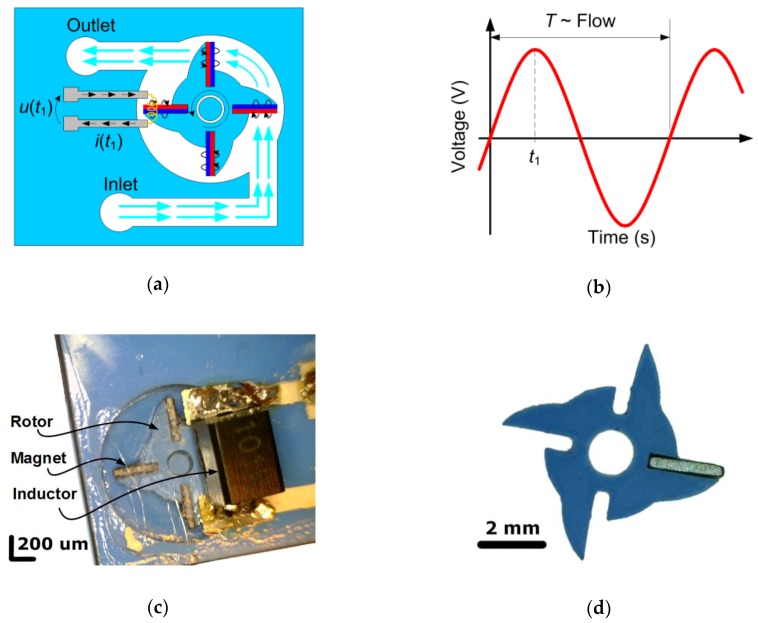
Electromagnetic flow sensor: (**a**) Principle of operation; *i*(*t_x_*), *u*(*t_x_*) current and voltage across the inductor; (**b**) Ideal waveform of output signal; *T*—period; *t*—time; (**c**) Flow sensor technological implementation; (**d**) Shape of turbine’s rotor.

In the proposed solution of the flow sensor with a turbine with magnets, the alternating current across a conductor is caused by the varying magnetic field. When gas in fluidic channels pushes wings of the rotor with fixed magnetic elements, the alternate current of frequency proportional to the volume flow rate is generated (Figure 2a). The permanent magnets have to be fixed to the subsequent rotor wings in the opposite way with regard to magnetic poles. Then the magnetic field with alternate direction is produced due to the rotation and the current of frequency *f_L_* = *N*·*RPS*/2 (where *N* is the number of wings and *RPS*—revolution per second) is generated in the measuring induction coil (Figure 2b).

### 3.2. Improvements in Technology

From a technological point of view, the novel LTCC turbine flow sensor consists of three main parts: a chamber, rotor, and cover. The LTCC chamber is equipped with the shaft for the rotor, the gas inlet, and the outlet (Figure 2c). The manufacturing process starts from lamination of 8 green DP951 (DuPont, Wilmington, DE, USA) tapes (254 µm), then the chamber is milled using a CNC routing machine. Such a process flow prevents any misalignment of tapes and provides very fine edges; the milling inaccuracy is lower than 50 µm. The obtained results are much better than what is reached in the typical LTCC process flow where single tapes are machined using a laser system and then stacked together; the stacking misalignment is in the range of around 100 µm. After thermal processing with using standard DP951 sintering profile, the chamber deepness is 0.84 mm. Then, the chamber is covered with a glaze layer and fired in a belt furnace. The glaze layer reduces the friction forces between the substrate and the rotor.

The vital part of the flow sensor is the rotor with permanent magnets connected to the wings (Figure 2d). The rotor is made from three thermo-compressed LTCC foils which were cut using an UltraViolet (UV) laser (here also stacking procedure was omitted). After firing, the permanent magnets were glued to the rotor wings. The main part of the flow sensor consisting of the rotor in the chamber and the discrete induction coil mounted on the top layer is presented in Figure 2c.

Several improvements according to the previous sensor versions [5,7] can be pointed out when considering the author’s technological process. First of all, the need to use any transparent glass or SVM materials as well as to execute precise alignment of stacked tapes or optical devices (diode and detector) are eliminated. Thanks to that, the accidental cases of rotor blocking inside chamber (which occurred in the previous Jurków’s solution [5]) are prevented.

### 3.3. Signal Conditioning Circuit

The signal generated by the sensor depends on the coil inductance and distance between the coil and the permanent magnets. Since these parameters are constant, the frequency and amplitude of the signal varies only with a value of rotation speed that is dependent on the flow rate. However, the signal amplitude is on the level of few mV in the input signal range from 1.5 to 10 liters per minute. This voltage is too low and too noised for efficient measurements of its frequency in a microprocessor system; thus, the voltage conditioning circuit is designed to improve the signal quality.

**Figure 3:**
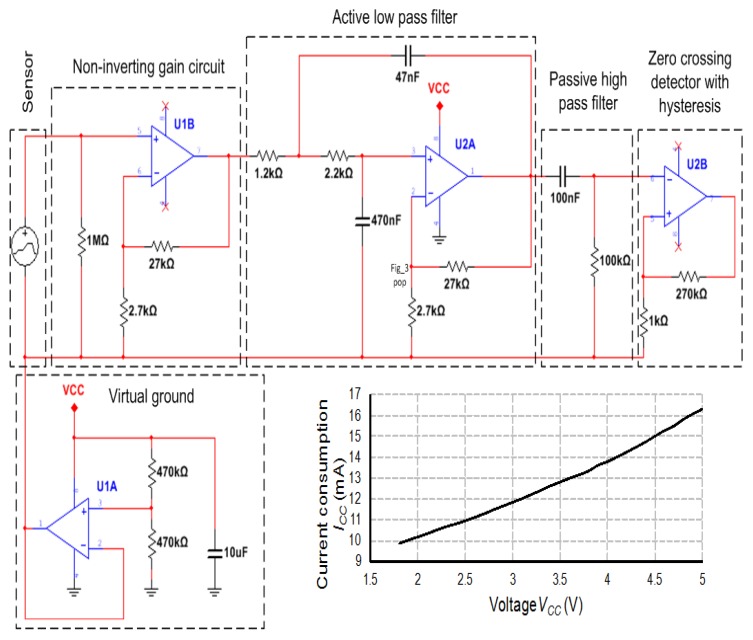
Block diagram of ultra low power signal conditioning circuit and diagram of current consumption as a function of supply voltage; *V_CC_*—voltage of power supply; *I_CC_*—current of power supply.

The signal conditioner (Figure 3) meets the requirements of ultra low power devices and hence the assumptions for the autonomous RFID transponder-sensor. Its construction is based on the energy efficient operational amplifier OA2NP (STMicroelectronics, Geneva, Switzerland) [39] that is characterized by operating voltage of 1.5 V–5.5 V, current consumption of 580 nA per channel, bandwidth of 8 kHz, voltage gain more than 30 dB in the operating frequency band up to 200 Hz, as well as rail-to-rail type of input/output. Symmetrical supply voltages of the amplifiers are provided thanks to a virtual ground stabilized by a voltage follower.

The first non-inverting amplifier with the voltage gain of 11 V/V is for signal preconditioning. Next, the second-order low-pass Sallen Key filter is used for attenuating noise and interferences (the 3 dB band is equal about 162 Hz). The third block, passive first-order high-pass filter solves the problem of passing Direct Current (DC) signal that could disturb the operation of a zero-crossing detector that is applied at a microcontroller input/output buffer.

Since microcontrollers are commonly equipped with Analog to Digital (A/D) converters, it obvious seems that analogue signals should be sampled and measured values should be determined digitally in the proposed autonomous sensor node. Unfortunately, the A/D blocks in microcontrollers consume significant amount of energy. For example, the supply current of a common A/D converter in an ultra low power chip is equal about 1.45 mA whereas processor core consumes only 0.34 mA [40]. For these reasons, the solution with zero-crossing detector (the estimated supply current of such a detector is equal about 1 µA) and period/frequency of the square wave measured by microcontroller timer/counter (the estimated supply current of the timer/counter block is equal to about 5 µA) is more energy efficient and is applied in the experimental circuit.

The frequency value can be obtained on the basis of indirect method consisting of measuring a single period of input signal. It involves only one counter that counts number *N* of clock pulses *T_C_* gated by the input signal. The period *T_X_* can be calculated as *T_X_* = *N·T_C_*. In order to increase the accuracy, the measurement procedure is repeated several times, and the results are averaged and then recalculated by a calibration curve to obtain the flow rate.

### 3.4. Measurement Stand and Tests

**Figure 4:**
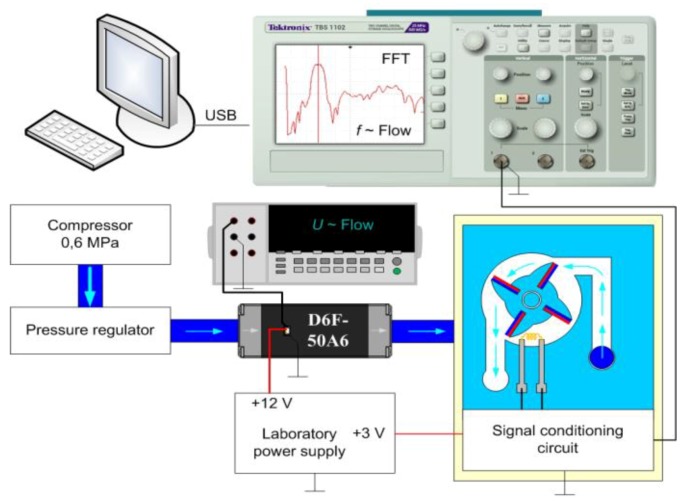
Block diagram of laboratory stand: D6F-50A6 is a reference flow meter. USB—Universal Serial Bus; FFT—Fast Fourier Transform; D6F-50A6—flow-meter of fluid (OMRON, Warszawa, Poland); *f*—measured frequency; *U*—measured voltage.

The calibration curve of the elaborated flow sensor has been determined in a special laboratory stand (Figure 4). A common compressor is used as the air stream source. Because of high operating pressure generated by the device, it is also necessary to use a control valve with smooth regulation of the air flow. Reference data for calibrating the developed sensor are obtained from the commercial flow-meter of fluid and a laboratory digital voltmeter. The OMRON D6F-50A6 (OMRON, Warszawa, Poland) device is used since it has a satisfactory accuracy of ±3 % and slight resistance to the fluid stream (1.44 kPa). Acquired waveforms at the output of the flow transducer or entire sensor are observed on the Tektronix TBS1102 (Textronic, Bracknell, UK) digital oscilloscope.

**Figure 5:**
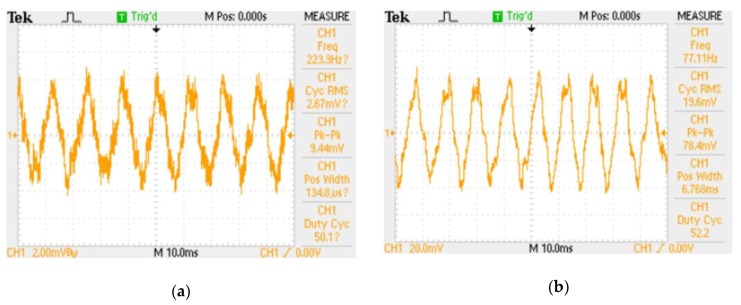
Waveforms at output of flow transducer: (**a**) Signal straight from output of flow transducer; vertical sensitivity in oscilloscope channel 2 mV/division; (**b**) Signal after amplification by active differential probe; vertical sensitivity in oscilloscope channel 20 mV/division.

A very important stage in designing the turbine flow sensor is to find out the conversion characteristic of the mechanical-to-electrical signal transducer. The frequency of the output signal is dependent on the rotational speed of the rotor and further on the intensity of the fluid flow. Additionally, it is strongly noised. Thus, the digital oscilloscope and dedicated firmware (OpenChoice Desktop, Textronic) are used to measure and collect waveforms. Preliminary experiments have shown an amplitude of about a few mV (Figure 5a). The maximal vertical sensitivity of the oscilloscope is 2 mV thus the active differential probe based on the AD620 (Analog Devices, Norwood, MA, USA) instrumentation amplifier is used in order to amplify the signal by 10 times (Figure 5b). On the basis of the obtained results, the operation characteristic of the transducer has been derived and is used at the stage of simulating (in electronic circuit simulator Multisim, National Instruments, Austin, TX, USA) and designing the signal conditioning block that is described in Section 3.3. The main goal of this block is to adjust the signal shape and level to the requirements valid at the input converter in the microprocessor unit. 

**Figure 6:**
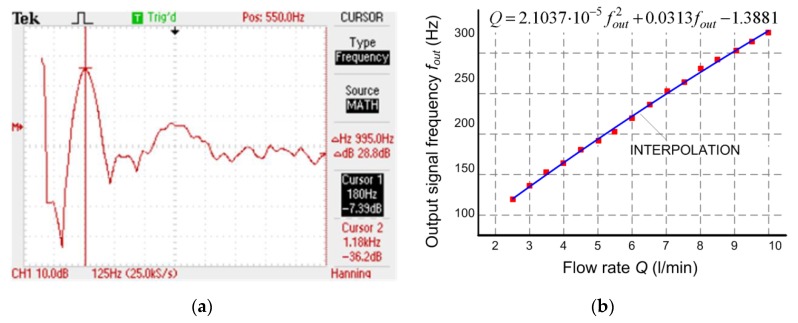
Sensor examination: (**a**) Averaged value of output signal spectrum at 5 l/min; (**b**) Calibration curve; *Q*—flow rate; *f_out_*—frequency of output signal.

Since the output signal from the rotation/frequency transducer is strongly disturbed by the noise, the special procedure of frequency measurements has to be applied. The measurement consists in determining fast Fourier transform after averaging 128 frames by using a digital oscilloscope (Figure 6a). The characteristic function of the sensor is the polynomial interpolation of obtained results (Figure 6b).

The comparison of earlier versions of optoelectronic LTCC turbine flow sensors and the proposed electromagnetic solution is presented in Table 2.

## 4. RFID Sensor with Two-Antenna System

### 4.1. The Second Harvester

In order to meet the project requirements, the designed full autonomous RFID transponder has to be equipped with an additional power source, besides the common harvester in the chip that gathers energy from the electromagnetic field generated by the RWD. It is obvious that almost in all typical environmental conditions there are active radio systems such as a mobile GSM or a WiFi wireless network and others. Unfortunately, the power harvesters providing energy from these sources are characterized by a very low voltage output and current efficiency [3]. It causes the necessity to use ultra low power Integrated Circuits (ICs) and passive elements with low quiescent current while internal electronic circuit is designed. Nevertheless, it should be mentioned that harvesting energy from radiocommunication systems (other than the RFID system) seems to be excellent solution regarding technological aspects of electrical circuit integrity. 

**Figure 7:**
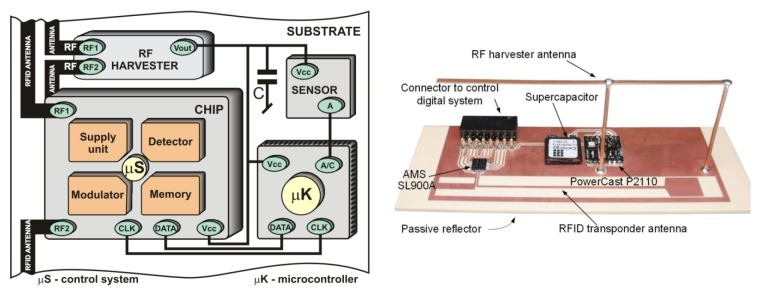
Autonomous RFID transponder: (**a**) Diagram of internal blocks; (**b**) Test model of autonomous transponder; RF—RadioFrequency; µS—digital control system; µK—microcontroller, C—supercapacitor; AMS SL900A—RFID chip (AMS AG, Premstaetten, Austria); P2110—RF harvester (Powercast, Pittsburgh, PA, USA); Vcc—voltage of power supply; CLK—clock of serial bus; DATA—data of serial bus; Vout—output voltage of RF harvester; A—analog output of sensor/transducer; A/C—analog-to-digital converter; RF1,RF2—inputs of antennas.

The example of full autonomous sensor with the RFID interface (Figure 7) that uses energy gathered only from the electromagnetic field is built on the basis of one of very rare commercial radiofrequency power harvester (RF harvester) dedicated to the band of 902–928 MHz (e.g., P2110; Powercast, Pittsburgh, PA, USA) [41]. The recovered energy stored in the supercapacitor Cellergy CLC03P025F12 (Cellergy, Migdal Haemek, Israel) with a capacity of 25 mF, maximal operating voltage of 3.5 V, and leakage current of 3 µA is utilized for supplying the sensor block with the low power microcontroller STM32L151RBT6 (STMicroelectronics, Geneva, Switzerland) and for activating memory in RFID chip outside the IZ. The RFID transponder is based on the AMS SL900A chip (AMS AG, Premstaetten, Austria) [42].

### 4.2. Two-Antenna System

**Figure 8:**
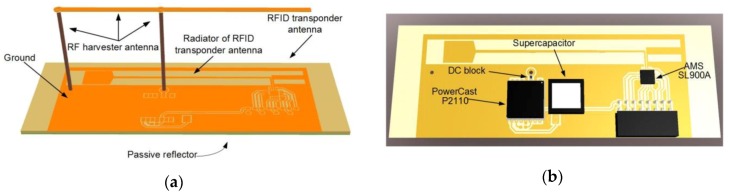

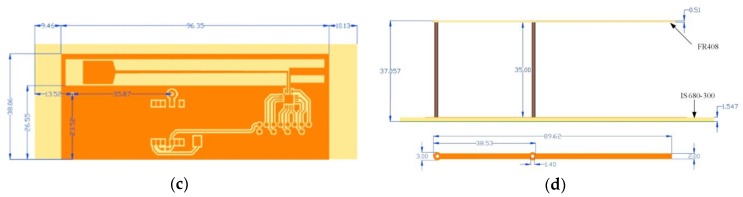
Elaborated model of two-antenna system: (**a**) Main components of model; (**b**) Electronic components taken in to consideration in simulation process; (**c**) Dimensions of RFID antenna (**d**) Dimensions of additional harvester antenna; FR408—high performance laminate (Isola Group, Düren, Germany); IS680-300—very low-loss laminate material (Isola Group).

In the designed autonomous RFID transponder, two antennas have to coexist in close proximity. The problem of their mutual interferences has to be overcome. The spatial antenna arrangement consists of two parts: the lower component serves as the transponder antenna, whereas the upper element of F-type (placed 35 mm above the substrate) harvests additional energy (Figure 8a). The radiating elements and all electrical tracks are surrounded by the ground plane that helps to immunize the electrical circuit against influences of the environment, also including structural material of substrates (identified objects). Under the RFID antenna on the bottom side, the passive reflector is created. It helps to form a radiation pattern and also separate the radiator from influence of an identified object. The possible impact of additional electronic circuit components is also taken into consideration in the simulation model (Figure 8b). Indicative dimensions of the antennas derived from numerical calculations in Mentor Graphics HyperLynx 3D EM (HL3DEM; Mentor Graphics, Wilsonville, OR, USA) software are highlighted in Figure 8c,d. 

**Figure 9:**
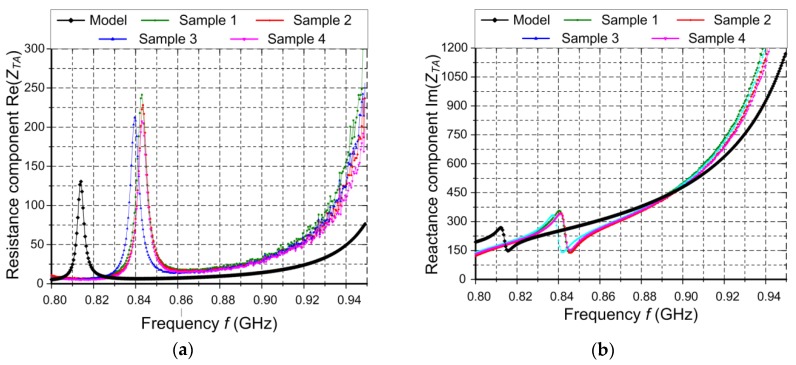
Impedance parameters of transponder antenna as a function of frequency *f*: (**a**) Resistance component; (**b**) Reactance component; *Z_TA_*—antenna impedance; Re—real part of complex number; Im—imaginary part of complex number; *f*—frequency.

**Figure 10:**
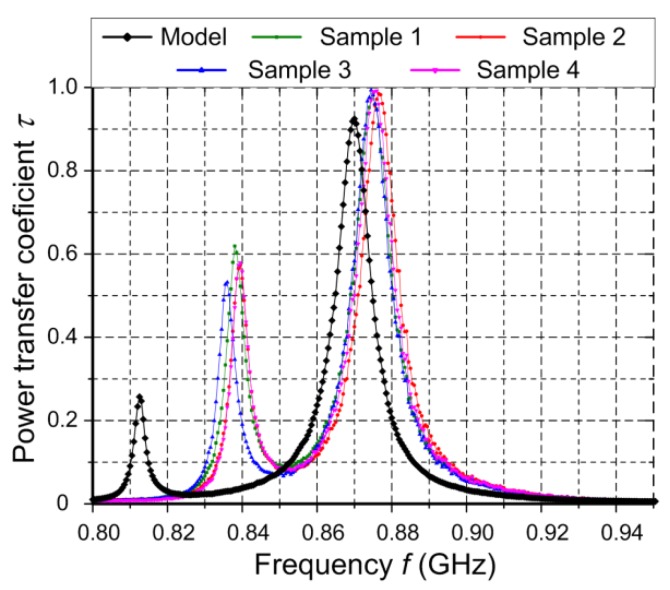
The coefficient of power transferring from antenna to the chip *τ* as a function of frequency *f*.

**Figure 11:**
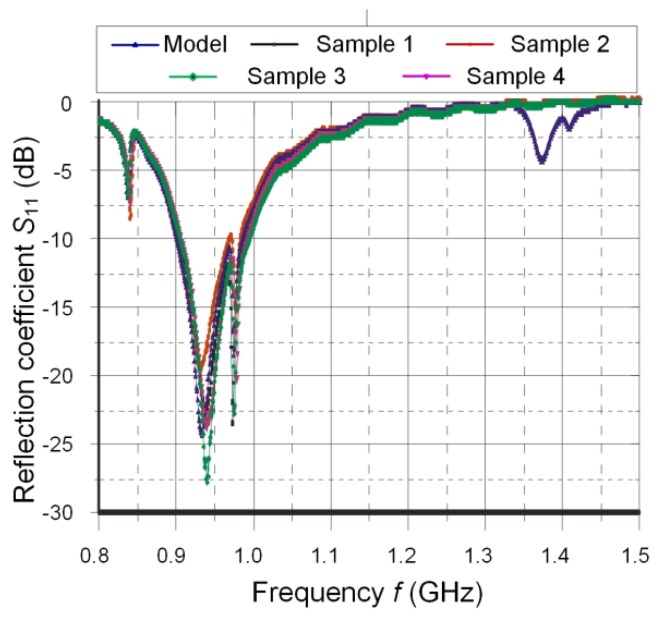
Reflection coefficient *S*_11_ as a function of frequency *f* for model and test samples.

Since the both antennas have diverse purposes, the verification process is carried out on the basis of totally different parameters. An antenna impedance (Figure 9) and a coefficient of power transfer (Figure 10) are quality factors for the RFID antenna whereas a reflection coefficient (Figure 11) has to be determined for the harvester antenna.

In the RFID transmission, the back-scattering modulation is used (in the transponder-RWD direction); besides the data, energy is also conveyed (in the RWD-transponder direction), and the chip input impedance strongly depends on the electromagnetic field strength. In consequence, the impedance parameters (Figure 9) and the coefficient of power transfer from antenna to the chip *τ* (Figure 10) are measured as quality factors of the RFID antenna. 

According to diagrams presented in Figure 9, the resonance frequency is higher than it results from the elaborated model, and the discrepancies between the measured and calculated impedance increase as the frequency value goes up. Nevertheless, it should be noted that these divergences appear outside the operating band. When comparing the impedances at operating frequency *f*_0_ = 866.6 MHz, the measured value is close to the one calculated on the base of the model (e.g., for the first sample *Z_TA_* = (17.72 + j296) Ω and the model *Z_TA_* = (7.84 + j329) Ω).

The second parameter *τ* represents the impedance matching between the antenna and chip (Figure 10). It can be calculated on the basis of dependency:

(1) τ(P)=4Re(ZTA)Re(ZTC(P))Re(ZTA+ZTC(P))2+Im(ZTA+ZTC(P))2,

where *Z_TA_* is the antenna impedance, *Z_TC_*(*P*), chip impedance with respect to a power dissipated on the load. As can be seen in Figure 10, convergence in the operating band is also high.

The additional energy harvester has the antenna input with the matched impedance of 50 Ω, and it can be supplied with a signal from −10 to +10 dBm. The highest efficiency of the P2110 module is in the frequency band of 902–928 MHz that covers Industrial, Scientific, Medical standard (ISM) but in North America (the P2110 is dedicated to this market). Nevertheless, it is also able to operate outside the main band with lower efficiency. The antenna is designed for a frequency range of 925–960 MHz; this band is reserved to the data and also energy transmission in the European GSM900 system (from a Base Transceiver Station (BTS) to mobile terminals). It has the construction of an inverted F-type and consists of a radiator, feed-line, and short-circuit stub. The additional capacitor (100 pF) is used for DC blocking because the antenna shorted to ground cannot be connected to the harvester radiofrequency input. The convergence of model and parameters of test samples is presented in Figure 11 on the basis of *S*_11_ reflection coefficient.

### 4.3. Verification

**Figure 12:**
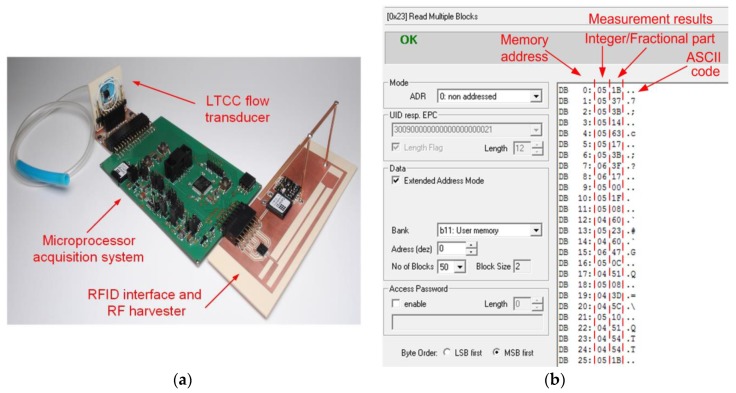
(**a**) Model of the autonomous RFID flow sensor; (**b**) The part of transponder memory with measured data read by RFID interface; ASCII—American Standard Code for Information Interchange; ADR—address; UID—Unique Identification number; EPC—Electronic Product Code; LSB—Least Significant Bit.

The functional tests of the model of autonomous semi-passive RFID transponder-sensor (Figure 12a) consist of performing measurements cyclically while the elaborated device is being powered from the electromagnetic field. The one cycle procedure consists of six steps: waking up microprocessor measuring unit, performing a series of 10 measurements of the signal frequency at the output of the flow sensor, averaging the results, calculating flow values, writing data to the RFID chip memory, introducing power down mode with activation of only the real time clock.

The measured flow rate values are stored as floating-point numbers in the memory of RFID chip. The microprocessor writes data to the chip by the SPI. The memory map consists of two-byte records: the integer and fractional part of a result. The acquired data can be read by the RWD when the autonomous RFID flow sensor appears in the IZ (Figure 12b).

The procedure of fluid flow measurement (frequency of measurement cycle and its duration) is strongly dependent on the amount of energy gathered by the RF harvester. The second factor that has a significant negative impact on the energy balance in the system is a leakage current of used supercapacitors. The microcontroller can be woken-up only when its power voltage reaches the operation level, which means that the electromagnetic field is so strong that the supply current is higher than all loads generated by passive and active elements in a quiescent state. Thus, in order to establish the exact time of fluid flow, measurement of the Real Time Clock (RTC) should be used. By adjusting time intervals between cycles of microcontroller activity, it is possible to affect the energy stability of the entire system. Further, since the RFID chip is equipped with an Electrically Erasable Programmable Read-Only Memory (EEPROM), a total collapse of the supply source does not destroy the data stored in it, and the information can always be accessed via the RFID interface. According to the ISO/IEC 18000-6c standard, the SL900A operates in accordance with the EPC Class 1 Gen 2. Its memory is divided into five banks: system, reserved, EPC value, TID unique identifier, programmed and locked during production with optional space for user data (8 416 bit). However, the limited capacity of about 8 kb has to be taken into consideration when the measurement procedure is designed, and the fact that the chip memory has a completely different addressing method under the RFID system than it is from the SPI side. To cope with the problem, the pointer of samples is created. It also allows avoidance of data overwriting.

**Figure 13:**
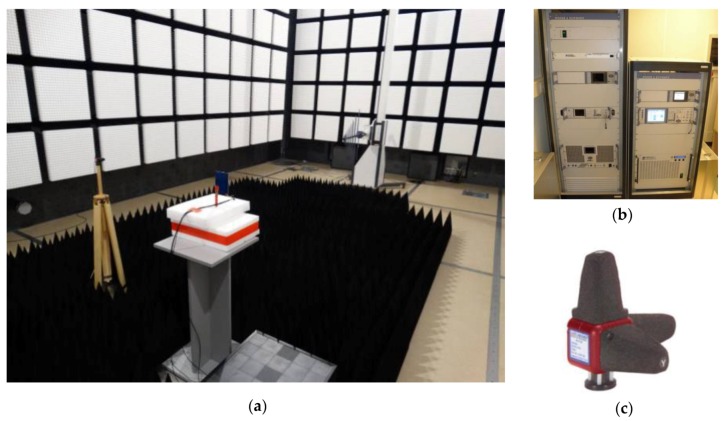
Verification stand (**a**) Test bed to analyze strength of the electromagnetic field in anechoic chamber TDK; (**b**) HI6005 Probe; (**c**) Generator and amplifier systems for generating electromagnetic waves in the range of up to 1 GHz and from 1 to 6 GHz.

Due to the applicable legal regulation regarding the use of frequency bands for radio transmission, all experiments related to testing the effectiveness of the developed flow sensor and its work in low energy conditions have to be carried out in an anechoic chamber. The measuring stand in the TDK chamber being the equipment of the authors’ RFID laboratory is presented in Figure 13a. The dedicated method elaborated to determine the electric field strength in the environment has to be used in order to check the device operational properties. It is based on the principle that the radiation pattern of the transmitting RWD antenna is symmetrical. It allows us to measure the electrical field strength and to harvest power in the sensor simultaneously, whereas the mutual influence on the electromagnetic field by the laboratory equipment and the device under test are eliminated. A set of apparatus for generating and measuring the electromagnetic field used in Electromagnetic Compatibility (EMC) research are adjusted to conduct the experiment (Figure 13b): SMB 100A generator, BBA 100 power amplifier, NRP2 power meter Rohde & Schwarz, HI6005 ETS-Lindgren (ETS-Lindgren, Mekaanikontie, Finland) probe (Figure 13c). The use of EMS32 Rohde & Schwarz firmware allows us to automate the acquisition process. 

**Figure 14:**
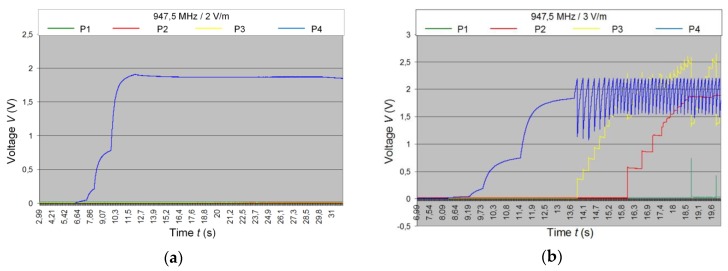
Voltage waveforms in P1-P4 test points for middle frequency of 947.5 MHz: (**a**) At field strength of 2 V/m; (**b**) At field strength of 3 V/m.

The research was conducted for 947.5 MHz middle value of the GSM900 frequency band (925–960 MHz). Voltage at four point of the tested sensor is highlighted in the Figure 14: P1—voltage at supercapacitor terminals; P2—voltage at capacitor in RTC supply circuit; P3—voltage at capacitor microcontroller supply circuit; P4—voltage at harvester output. The flow RFID sensor works performing the measuring and data storing algorithm in pulse regime with 60 s time interval cycles.

The developed RFID flow sensor starts to work at 3 V/m strength field. However, if the supercapacitor with lower leakage current, as well as the microprocessor system better adjusted to requirements and tasks (microcontroller was chosen with regard to possibility of conducting more complicated algorithms in order to support wide spectrum of research) of the designed device were used, the energy balance would be significantly improved. Then, the sensor could operate in a weaker electromagnetic field.

**Figure 15:**
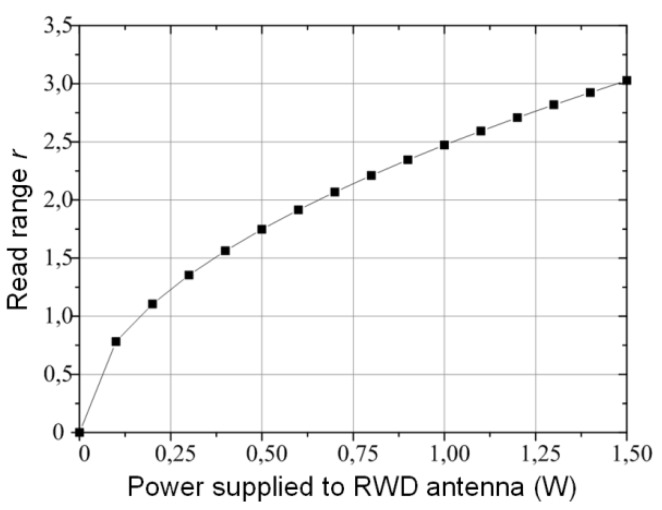
Tests of RFID interface in flow sensor—read range *r*.

The efficiency of the developed flow sensor was also tested in the RFID system by determining a read range *r* parameter (Figure 15). The RFID interface was checked in the Authors’ RFID laboratory according to accomplished requirements of electronic product code in the UHF band (protocol ISO 18000-63, RWD compatibility European version of ETSI EN 302 208—2 W ERP, frequency band 865.6–867.6 MHz).

The ID ISC.LRU2000 (Feig Electronic, Weilburg, Germany) long-range read/write device with ID ISC.ANTU250/250 antenna is used in the experimental test bed. The main advantage of this device is the possibility to set up communication protocol parameters according to investigators’ tasks. Also, the level of output power can be adjusted in the development software (ISOStart 2011 v. 8.03.02, Feig Electronic). The RFID interface performance can be estimated from the diagram of read range changes as a function of the power supplied to the RWD antenna that is presented in Figure 15.

## 5. Conclusions

The authors have proposed the unique construction of an autonomous RFID flow sensor that is dedicated to operating as a wireless sensor node. The elaborated device can operate not only in the RWD presence, but also outside the IZ without any supply by chemical cells. Additionally, the authors improved LTCC flow sensor with respect to the energy balance that allows development of the construction dedicated to work in harsh environments. 

The proposed construction of autonomous RFID transponder-sensor gains benefits from the author’s achievements in the LTCC technology, RFID technique, and world progress in low power electronics. This paper shows that even at the contemporary stage of electronics development, it is possible to create an autonomous battery-free device with RFID interface, in which energy is harvested from the electromagnetic field that are available in the application environment. Taking into account permanent progress in the electronics and regarding technological possibilities to integrate low power transducers in technologies used in the RFID technique, it can be predicted that such devices will be accessible in the commercial market in the close future.

## Figures and Tables

**Table 1:** Comparison of RadioFrequency Identification (RFID) transponder types. IZ—Interrogation Zone; PT—Passive Transponders, S-PT—Semi-Passive Transponder, SENSOR—Semi-Passive Transponder-Sensor.

Type of RFID Transponder	Activity in IZ	Activity outside IZ	Abbr. in Figure 1
Passive transponders	Only identification	No active	PT
Semi-passive transponder	Identification and data acquisition	Data acquisition with battery	S-PT
Semi-passive transponder-sensor	Identification and data acquisition	Data acquisition without battery	SENSOR

**Table 2:** Parameter comparison of Low Temperature Co-fired Ceramic (LTCC) turbine-based flow sensors.

Parameter	Sensor Presented in [6]	Sensor Presented in [5]	The Author’s Flow Sensor
Range of Input Signal	6–17 ml/s (0.36–1 l/min)	7–25 ml/s (0.42–1.5 l/min)	25–167 ml/s (1.5–10 l/min)
Range of Output Signal	70–420 Hz	120–330 Hz	110–300 Hz
Operating Pressure	<150 kPa	<150 kPa	<60 kPa
Current Consumption	4.6–6.2 mA (optical elements + signal conditioner)	4.6–6.2 mA (optical elements + signal conditioner)	10–16 µA (generator of minute voltage + signal conditioner)
Technological Inaccuracy	~100 µm stacking misalignment	~100 µm stacking misalignment	~50 µm milling inaccuracy
